# Metabolomic and phenotypic implications of the application of fertilization products containing microcontaminants in lettuce (*Lactuca sativa*)

**DOI:** 10.1038/s41598-021-89058-x

**Published:** 2021-05-06

**Authors:** Víctor Matamoros, Alicia María Rendón-Mera, Benjamí Piña, Đorđe Tadić, Núria Cañameras, Nuria Carazo, J. M. Bayona

**Affiliations:** 1 Department of Environmental Chemistry, Institute of Environmental, Assessment and Water Research (IDAEA), Spanish National Research Council (CSIC), c/Jordi Girona, 18-26, 08034 Barcelona, Spain; 2 Grupo GDCON, Facultad de Ingeniería, Sede de Investigación Universitaria (SIU), Universidad de Antioquia, Calle 70 # 52-21, Medellín, 050010 Colombia; 3 Department of Agri-Food Engineering and Biotechnology (DEAB), Polytechnic University of Catalonia (UPC), Esteve Terrades 8, Building 4, Castelldefels, Spain

**Keywords:** Metabolomics, Plant stress responses, Environmental sciences, Environmental chemistry

## Abstract

Cultivation practice using organic amendments is plausible to ensure global food security. However, plant abiotic stress due to the presence of metals and organic microcontaminants (OMCs) in fertilization products cannot be overlooked. In this study, we monitored lettuce metabolism and phenotypic response following the application of either sewage sludge (SS), the organic fraction of municipal solid waste, swine manure (SM), chemical fertilizers (CF), or no amendment (C) in a greenhouse facility. The experimental set-up consisted of five treatments with five replicates (25 experimental units randomly distributed). All fertilizers were supplied at the equivalent agronomic total nitrogen dose, but the occurrence of trace metals and/or OMCs was greater in the SS and SM than the rest. Non-target metabolomic analysis (high-resolution mass spectrometry coupled with partial least squares regression) identified more than 300 plant metabolites (amino acids, organic acids, sugar alcohols, and sugars), 55 of which showed significant changes in their relative abundances depending on the type of amendment. Functional analysis indicated that the use of CF or SS increased the levels of metabolites involved in carbohydrate and nitrogen metabolism. Therefore, although SS and SM fertilizers had a greater presence of heavy metals and/or OMCs, our results indicate that they did not induce measurable adverse effects in the lettuce phenotype or metabolism. Metabolic changes between fertilizers (CF and SS *vs.* C and SM) were mainly due to nitrogen availability.

## Introduction

According to the latest United Nations projections, food production will have to increase 70% to meet the global food demand by 2050^1^. In this regard, wastewater reuse and organic waste amendments offer alternative water and fertilization sources in the context of the circular economy and sustainable agriculture. The current situation will be exacerbated by the fact that soils have lost organic matter, as well as water- and nutrient-holding capacity, due to intensive arable farming activity^2^. Soil structure and quality plays a critical role in both crop productivity and soil and crop resilience to drought and flood events^3^. It is thus necessary to promote soil conservation and find sustainable fertilization practices.

Different organic wastes can be used to solve this issue. Among them the most employed fertilization products are: animal-based waste (manure), compost (plant sources or food waste), and urban waste (sewage sludge and household waste)^4^. Manure has been used at farms across the planet for centuries. The organic fraction of municipal solid waste (OFMSW) refers to a mixture of waste from parks, gardens, and kitchens that can be transformed it into usable compost^5^. OFMSW is rich in organic components such as carbohydrates, lipids, proteins, and organic acids, making it an excellent source of fertilization^6^. Sewage sludge (SS) is a form of organic waste rich in nutrients, among them phosphorus. Nevertheless, some of these fertilization products may pose a potential human health risk if they are used in agriculture, since they contain different chemical substances. These chemical substances include trace elements, such as Zn, Cd, Cu, Ni, and Cr, as well as organic microcontaminants (OMCs), such as contaminants of emerging concern (CECs), albeit at lower concentrations. For example, swine manure (SM) contains high concentrations of antibiotics and antibiotic resistance genes^7,8^, while SS contains metals and CECs such as pharmaceuticals or personal care products^9,10^, and OFMSW may contain microplastics from municipal waste^11^.

Current studies suggest that the presence of metals or CECs in agriculture may have the potential to change both crop morphological (e.g., biomass production and shoot growth) and physiological (e.g., phytohormones and chlorophyll content) profiles^12^. For example, Wang et al.^13^ demonstrated that exposure of radishes under hydroponic conditions to Pb stress at high concentration levels (1000 mg L^−1^) results in profound biochemical changes including to carbohydrate metabolism, energy metabolism, and glutathione metabolism, while the addition of Cd (400 mg L^−1^) causes significant variations in energy production, amino acid metabolism, and oxidative phosphorylation-related pathways. Hurtado et al.^14^ observed that exposure of lettuce plants to CECs through water irrigation at environmentally relevant concentrations can change the plant phenotype and cause significant metabolic alterations in the plants (carbohydrate metabolism, citric acid cycle, pentose phosphate pathway, and glutathione pathway). Nevertheless, although there is information about the plant uptake of these substances from different organic fertilizer amendments^10^, there is no evidence of crop phenotype and metabolism changes following fertilization amendments.

The present study aims to assess the effect of different fertilization products (SS, OFMSW, and SM) on lettuce morphology and metabolism and to compare these results with those for lettuces exposed to chemical fertilizers (CF). The hypothesis of the study is that the application of different fertilization products can result in lettuce metabolic and phenotypic changes due to the presence of metals and CECs.

## Results and discussion

### Characterization of fertilization products and plant uptake of metals and OMCs

Table 1 shows the chemical compositions of the different soil amendment fertilizers assessed in this study. Although the total nitrogen (Kjeldahl) composition was similar among all the fertilization products, ammoniacal-N, and P were statistically greater in the SS fertilizer. Nevertheless, the SS fertilizer also had the highest concentration of heavy metals (statistically significant for Cu, Cr and Ni) (Table 2). Specifically, Cu and Zn had the greatest concentration levels. These concentrations are consistent with those found in other studies conducted around the world^15,16^ and are below the threshold values set in Spanish Royal Decree 1320/1990 and the EU rules (PE-CONS 76/18) for the use of fertilization products in agriculture. Despite the greater concentrations of metals in the SS fertilizer (Table 2), the concentration levels of metals were greater in lettuces amended with SM and OFMSW. This suggests that the organic matter composition of the SS had a greater interaction with these chemical susbtances and, therefore, lower plant uptake^17^. The concentration values of metals found in lettuces were below the levels set by the European Commission regulation No. 181/2006.

**Table 1:** Chemical composition in dry weight of organic fertilizers (n  = 3). Samples were analysed in triplicate. Different lowercase letters indicate statistically difference (*p* < 0.05). The amount of fertilization product added per pot was calculated to ensure the same quantity of total nitrogen in all treatments (100 kg of N per ha).

	SS	SM	OFMSW	CF*
Kjeldahl Nitrogen (g kg^−1^)	27.7 ± 4.4^a^	22.9 ± 3.7^a^	26.1 ± 4.2^a^	340
Ammonia–nitrogen (g kg^−1^)	13.4 ± 0.9^a^	5.6 ± 0.5^b^	2.9 ± 0.2^b^	170
Nitrate-nitrogen (g kg^−1^)	< 0.1	0.2 ± 0.1	0.4 ± 0.1	170
P (g kg^−1^, acid extraction)	15.5 ± 6.2^a^	6.1 ± 2.1^b^	5.5 ± 2.2^b^	190
K (g kg^−1^, acid extraction)	21.0 ± 5.5^a^	8.2 ± 2.1^b^	24.2 ± 6.3^a^	430
Moisture content (%)	80%	70%	23%	-

*Chemical fertilizer composition was as follows: ammonium nitrate (34% N), phosphate (44% P_2_O_5_) and potassium sulfate (52% K_2_SO_4_).

**Table 2:** Concentrations of metals (in mg kg^−1^) and most frequently detected OMCs (in µg kg^−1^) in fertilization products (n = 3) and lettuce samples (n = 5). Results are shown in dry weight (dw) for fertilisers and in fresh weight (fw) for lettuce crops. Different lowercase letters indicate statistically difference (*p* < 0.05).

Compound	SS		SM		OFMSW		CF	
	Fertilizer	Lettuce	Fertilizer	Lettuce	Fertilizer	Lettuce	Fertilizer	Lettuce
Cd (mg kg^−1^)	0.51 ± 0.14	< 0.02	< 0.50	< 0.02	< 0.50	< 0.02	< 0.5	< 0.02
Cu (mg kg^−1^)	240 ± 60^a^	0.9 ± 0.4	72 ± 18^b^	1.3 ± 0.5	63 ± 16^b^	1.5 ± 0.6	< 0.5	0.6 ± 0.3
Cr (mg kg^−1^)	57 ± 19^a^	0.05 ± 0.02	10 ± 4^b^	0.16 ± 0.08	15 ± 5^b^	0.15 ± 0.09	< 0.5	0.02 ± 0.01
Ni (mg kg^−1^)	53 ± 12^a^	0.04 ± 0.01	3 ± 1^b^	0.09 ± 0.02	9 ± 2^b^	0.20 ± 0.10	< 0.5	0.04 ± 0.01
Pb (mg kg^−1^)	29 ± 10^a^	0.01 ± 0.01	3 ± 1^b^	0.02 ± 0.01	30 ± 11^a^	0.02 ± 0.01	< 0.5	0.01 ± 0.01
Zn (mg kg^−1^)	699 ± 105^a^	3.4 ± 1.5	535 ± 80^a^	5.4 ± 1.4	159 ± 24^b^	3.9 ± 1.1	< 0.5	2.8 ± 1.2
8-hydroxyquinoline*	149 ± 27	–	3397 ± 604	–	–	–	–	–
Lincomycin	25 ± 6	1 ± 1	9831 ± 2200	1 ± 1	–	–	–	–
Sulfacetamide	–	–	3.9 ± 0.9	–	–	–	–	–
Sulfadiazine	–	–	11 ± 2	–	–	–	–	–
Sulfathiazole	203 ± 12	–	–	–	–	–	–	–
Tetracycline	169 ± 33	–	5.9 ± 1.2	–	–	–	–	–
Oxytetracycline	–	–	918 ± 222	–	–	–	–	–
Ciprofloxacin	9317 ± 2600	14 ± 6	–	–	–	–	–	–
Ofloxacin	–	–	12 ± 2	–	–	–	–	–
Chlortetracycline	–	–	2.9 ± 0.6	–	–	–	–	–
Azithromycin	165 ± 51	3 ± 2	6912 ± 2100	–	1 ± 1	–	–	–

–:Non detected.

*Intermediate in the degradation of several antibiotics. REGULATION OF THE EUROPEAN PARLIAMENT AND OF THE COUNCIL laying down rules on the making available on the market of EU fertilizing products (PE-CONS 76/18. 2019) stablished a threshold values of metals in fertilizers as follows: Cd 1.5 mg kg^−1^; Cu 300 mg kg^−1^; Cr 100 mg kg^−1^; Ni 50 mg kg^−1^; Pg 120 mg kg^−1^, and Zn 800 mg kg^−1^. Statistical analysis were conducted using SPSS 25 software.

The untargeted LC-Orbitrap analysis revealed that the SS fertilizer had the greatest frequency of detection of OMCs (parent compound and several fragments), with 28 identified compounds (human pharmaceuticals and pesticides) out of 1298 compounds included in the suspected list (see SM section). It was followed by the SM, with 16 compounds (veterinary pharmaceuticals, hormones, and pesticides), and the OFMSW, with 3 compounds (household products) (see Supplementary Table S2 online). The number of detected compounds in the SS samples was consistent with previously published studies^18,19^ due to the wide spectrum of contaminants expected to be present in wastewater. Likewise, the veterinary pharmaceuticals detected in the SM samples were consistent with those previously reported for swine-slurry samples^20^. In contrast, only 3 of the 1298 compounds included in the suspected list were detected in the OFMSW samples. Since the most frequently detected OMCs in all fertilization products were veterinary pharmaceuticals (antibiotics), we did their quantification in fertilization products as well as in crops. Table 2 shows that the greatest concentration of antibiotics was found in SM, followed by SS, whereas antibiotics were not detected in OFMSW nor in the CF. Among the 11 antibiotics detected in the SS and SM (ranging from 250 to 9800 ng/g fw), only 2 were detected in crops (0.7–14 ng/g fw). This agrees with the fact that the presence of organic matter reduces the plant bioavailability of OMCs and metals^21^.

To sum up, SS and SM fertilizers contained the greatest concentration levels of studied pollutants (metals and/or OMCs), but their plant uptake was very limited. Our results are in line with other studies that suggest that the addition of fertilization products results in changes in soil properties such as organic matter content, pH or clay percentage, reducing the plant uptake/bioavailability of metals and OMCs^22^. Further details on the plant uptake and human health risk assessment of the occurrence of metals and OMCs in lettuces following soil amendment with these fertilization products are found in Margenat et al.^23^.

### Impact on plant phenotype

**Figure 1:**
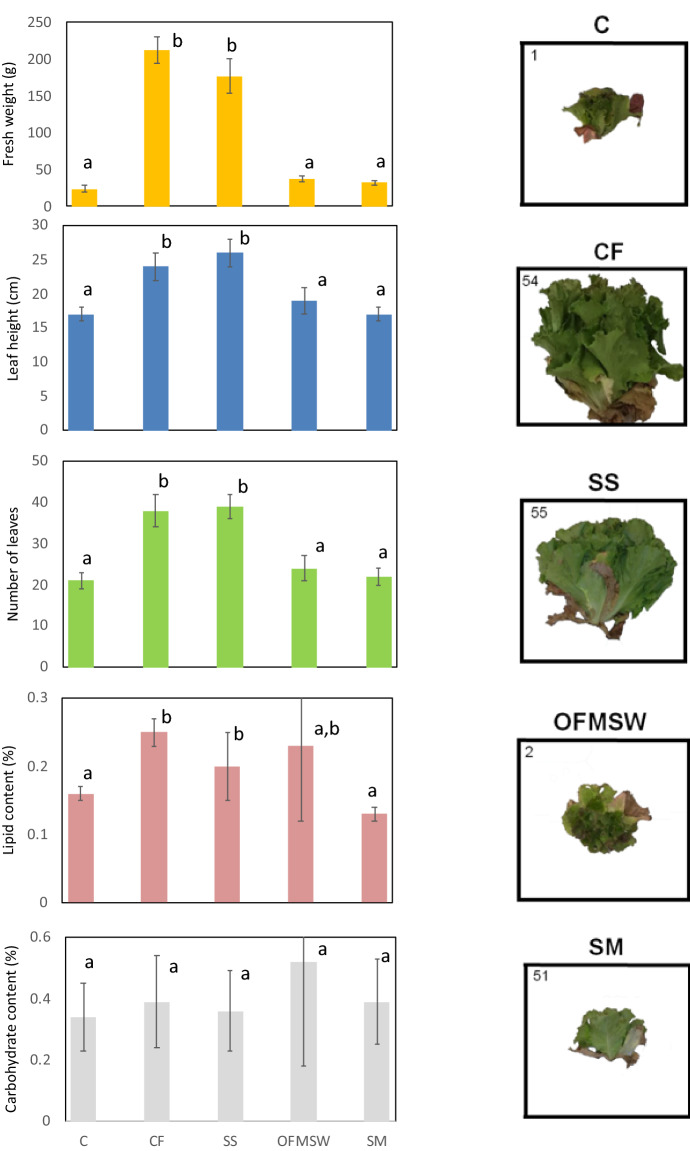
Phenotypic parameters of the lettuce plants grow under the different organic fertilizers, including control without fertilization (C) and control with chemical fertilization (CF) (n = 5). Different letters between treatments in the plots indicate statistical differences (*p* < 0.05). Pictures of the lettuce plants grown on the different organic fertilizers, including C and CF, and the following organic amendments. SS: sewage sludge; OFMSW: organic fraction of municipal solid waste; and SM: swine manure. Plot was created with Microsoft Excel (version16.6.6, https://www.microsoft.com/).

The application of the different organic fertilizers resulted in different phenotype changes (Fig. 1). Whereas the SS showed similar morphological parameters (i.e., fresh weight, leaf height, number of leaves) to those observed in the CF, the other organic fertilizers (OFMSW, SM, and C) resulted in lower morphological values (Fig. 1, *p* < 0.05). This is consistent with the fact that SS fertilizers had the greatest content of ammoniacal N and phosphorus among organic fertilizers (Table 1). In fact, the ammoniacal N form is an easily available source of N, which plants need to grow^24^, whereas a low P status has been tied to changes in the relative growth of roots and shoots, including decreased foliar area^25^. Nitrate–N concentration was greater in the OFMSW, but the concentration was very low in comparison to ammoniacal-N, so it is not expected to play an important role in the fertilization of lettuce crops.

No differences were observed in the carbohydrate content, but the lipid content followed the same trend as the changes in the morphological parameters (except for OFMSW which did not show differences). The results thus indicate that SS fertilization had a similar agronomic yield to the application of the CF, while the other treatments exhibited lower values. Therefore, our results suggest that the greater abundance of OMCs and heavy metals in the SS amendment (Table 2 and Supplementary Table S2 online) did not produce any effect on plant phenotype (Fig. 1). This is in partial disagreement with our previous studies in which the presence of OMCs in irrigation water modified plant morphology (leaves height and stem width)^14^. Nevertheless, in the current study we used real soil amended with fertilization products and therefore greater organic matter content, whereas the previous study was performed in a sandy soil with low organic matter content. Furthermore, and as it has been mentioned above, the plant uptake of metals and antibiotics in lettuces was very limited^23^. Therefore, the main explanation for the phenotype difference between SS and the other amendments may be nitrogen availability^24^. In fact, a lack of synchronization between nitrogen mineralization and nitrogen demand has been described as a challenge in culture strategies using fertilization products^26^. Therefore, though the fertilization products were applied ensuring a similar amount of Kjeldahl nitrogen, only ammoniacal nitrogen and nitrate nitrogen are easily available to plants. This is in agreement with the greater concentration of ammoniacal-N in the CF and SS treatments (Table 1), as well as with the fact that organic nitrogen was the predominant nitrogen form in the OFMSW and SM fertilizers^27^, and nitrate-nitrogen content was very low in all fertilization products.

### Correlation of phenotypic changes with metabolite profiling

Table 3 shows the VIP scores of the various aforementioned phenotypic parameters for each metabolite with a value greater than 1.5 (for at least one of the assessed parameters, i.e., 55 metabolites). The results show that 38 of the 55 metabolites are positively related (VIP scores > 1) to phenotypic plant characteristics (fresh weight, leaf height, and number of leaves). They include amino acids (aspartic acid, valine, proline, and phenylalanine), organic carboxylic acids (e.g., glyceric acid, hexanedioic acid, aminobutanoic acid, and butenedioic acid), sugar alcohols and others (e.g., tetrahydrolinalool, glycine, allylamine, and myoinositol), and sugars (e.g. arabinose, fructose, sorbose, tagatose, xylopyranose, mannose, allose, rhamnose, sorbofuranose, and ribofuranose). These results suggest that these compounds are associated with plant growth and that their accumulation probably requires significant amounts of energy. This is consistent with the studies performed by Telebi et al.^28^, who found that both the plant height and peduncle length of *Gazania rigens* L significantly increased following a foliar application of amino acids^29^. Arginine, phenylalanine, and valine have been correlated with photo assimilate storage^30^. Finally, the fertilization of plants with a high proportion of nutrients in solution has been observed to increase glucose and fructose concentrations in lettuce leaves^31^. Similarly, Kamenicka et al.^32^ observed that fructose, mannose, and xylose are the most effective carbon sources for shoot proliferation in saucer magnolia.

**Table 3:** VIP scores showing the correlation of the metabolites *vs.* agronomic parameters (PLS analysis). VIP scores > 1.5 were considered. ^a^Isomers, same mas spectrum. VIP scores > 1 are shown in bold. Unknown metabolites (RI/SI < 500) are not included. Statistical analysis were conducted using XLSTAT software.

Family	Match result name	Fresh weight	Leaf height	Number of leaves	Lipids	Carbohydrates
Aminoacid	Valine	**1.6**	1.2	1.3	0.1	0.5
Aminoacid	L-Proline	**1.5**	**1.3**	**1.3**	0.3	0.6
Aminoacid	Aspartic acid	**1.6**	**1.2**	**1.4**	0.2	0.5
Aminoacid	L-Phenylalanine	**1.7**	**1.4**	**1.6**	0.2	0.9
Aminoacid	Arginine	**1.8**	**2.0**	**2.0**	0.4	1.1
Organic acids	Phosphate	0.9	0.6	0.4	**1.3**	**2.7**
Organic acids	Retinoic acid	0.1	0.0	0.1	0.1	**1.5**
Organic acids	Glyceric acid	**1.8**	**1.9**	**1.9**	0.2	0.9
Organic acids	2-Butenedioic acid (fumaric acid)	**1.4**	**1.5**	**1.4**	**1.2**	0.1
Organic acids	Octanoic acid, cyclobutyl ester	**1.8**	**2.1**	**1.9**	0.3	0.6
Organic acids	4-Aminobutanoic acid	**1.6**	**1.2**	**1.3**	0.5	0.5
Organic acids	Pentanedioic acid (glutaric acid)	**1.6**	**1.5**	**1.5**	**1.7**	**3.3**
Organic acids	Tartaric acid	**1.1**	**1.1**	**1.2**	**2.0**	**1.4**
Organic acids	Succinic acid, ethyl 4-methylhept-3-yl ester	**1.4**	**1.2**	**1.3**	**1.1**	**2.0**
Organic acids	Quinnic acid	0.6	0.7	0.6	0.5	**2.5**
Organic acids	Ribonic acid	**1.2**	**1.1**	**1.0**	**1.5**	**1.3**
Organic acids	Methylglutaconic acid	**1.5**	**1.5**	**1.3**	0.7	**1.3**
Organic acids	Dehydroabietic acid	0.4	0.1	0.1	**2.0**	0.2
Organic acids	Hexanedioic acid, a-keto	1.4	1.2	1.4	**2.5**	0.0
Organic acids	Glucuronic acid, 6-lactone	**1.6**	**1.3**	**1.6**	**1.7**	0.2
Organic acids	Galactaric acid	0.4	0.4	0.5	0.6	1.5
Organic acids	Citric acid	**1.2**	**1.1**	**1.0**	0.9	0.4
Organic acids	Ketoglutaric acid	**1.2**	**1.0**	**1.1**	**2.4**	0.9
Organic acids	Gluconic acid	**1.5**	**1.3**	**1.5**	**1.9**	**1.9**
Sugar alc. and others	Tetrahydrolinalool, isomer 1	**1.8**	**2.2**	**2.0**	0.1	0.5
Sugar alc. and others	Tetrahydrolinalool, isomer 2	**1.8**	**2.2**	**2.0**	0.1	0.5
Sugar alc. and others	Glycine	**1.8**	**1.9**	**1.9**	0.2	0.8
Sugar alc. and others	Uridine	1.0	0.8	0.8	**1.1**	**1.5**
Sugar alc. and others	Propanetriol, 2-methyl-	**1.3**	**1.3**	**1.2**	**1.5**	0.0
Sugar alc. and others	Tri(n-butyl)difluorophosphorane	0.7	0.8	0.6	**1.4**	**2.3**
Sugar alc. and others	Inose, 2-desoxy-	0.2	0.3	0.1	**2.0**	0.8
Sugar alc. and others	Myo-inositol, isomer 1	**1.3**	**1.1**	**1.4**	**1.6**	**2.0**
Sugar alc. and others	Myo-inositol, isomer 2	0.2	0.1	0.2	0.6	1.0
Sugar alc. and others	Adenosine	**1.0**	**1.0**	**1.3**	**1.1**	0.3
Sugars	Arabinose	**1.7**	**1.4**	**1.7**	0.3	0.3
Sugars	Methyl α-D-ribofuranoside	**1.7**	**1.5**	**1.7**	**1.7**	0.3
Sugars	Tagatofuranose	0.5	0.5	0.5	**1.7**	0.8
Sugars	Galactose ethoxyme	1.0	0.8	0.8	**2.5**	0.6
Sugars	Galactofuranose, 2,6-di-O-methyl	0.6	0.5	0.3	**2.4**	**1.9**
Sugars	Fructose	**1.6**	**1.5**	**1.4**	**1.4**	**1.7**
Sugars	Tagatose	**1.8**	**1.7**	**1.6**	**1.3**	**1.6**
Sugars	Sorbose	**1.8**	**1.6**	**1.7**	**1.5**	**1.6**
Sugars	Galactose oxime	0.8	0.9	1.2	0.0	0.5
Sugars	1,2-O-Isopropylidene-α-D-glucofuranose	**1.5**	**1.4**	**1.3**	**1.1**	**1.6**
Sugars	Mannose	**1.7**	**1.6**	**1.5**	0.9	**1.6**
Sugars	Xylopyranose	**1.7**	**1.6**	**1.5**	0.6	1.6
Sugars	Allose,	**1.8**	**2.1**	**2.0**	0.2	0.4
Sugars	Glucopyranose	0.3	0.2	0.3	0.6	0.6
Sugars	Rhamonose	**1.3**	**1.2**	**1.0**	**1.8**	**1.7**
Sugars	Sorbofuranose	**1.0**	0.8	**1.0**	0.1	1.1
Sugars	Psicofuranose	0.9	0.7	1.0	0.2	1.2
Sugars	L-Rhamnose	0.5	0.7	0.8	0.8	**1.5**
Sugars	Maltose, isomer 1	0.1	0.0	0.1	**1.0**	0.4
Sugars	Sucrose	0.2	0.1	0.2	0.9	**2.0**
Sugars	Manobiose	0.1	0.2	0.5	0.8	**1.5**
Sugars	Ribofuranose	**1.3**	**1.2**	**1.4**	0.8	**1.7**

Conversely, lipids and carbohydrate content behaved differently. Acid compounds (phosphate, tartaric acid, ribonic acid, ketoglutaric acid, pentanedioic acid, retinoic acid, quinic acid and gluconic acid), organic alcohols (inose, myoinositol), and sugars (maltose, tagatofuranose, galactose, galactofuranose, glucopyranose, gluconic acid, mannobiose, rhamnose, sucrose) had a strong relationship with lipid and carbohydrate content, but a low or moderate one with morphological parameters. This relationship is consistent with the hypothesis that the plants that showed low growth were exposed to nutrient stress (nitrogen availability). In fact, quinic and tartaric acid derivatives have been described as the main up-regulated phenolic acid components in stressed plants^33,34^. In previous studies, we also observed increased quinic, ribonic, and tartaric acid content in lettuce plants exposed to CECs^14^. Similarly, growth development was observed to be related to myoinositol content. The regulation of myoinositol levels is critical to maintaining ascorbic acid^35^, phosphatidylinositol, and ceramide levels, which regulate growth, development, and cell death in *Arabidopsis thaliana*^36^.

### Metabolic response of lettuce to fertilizers

**Figure 2:**
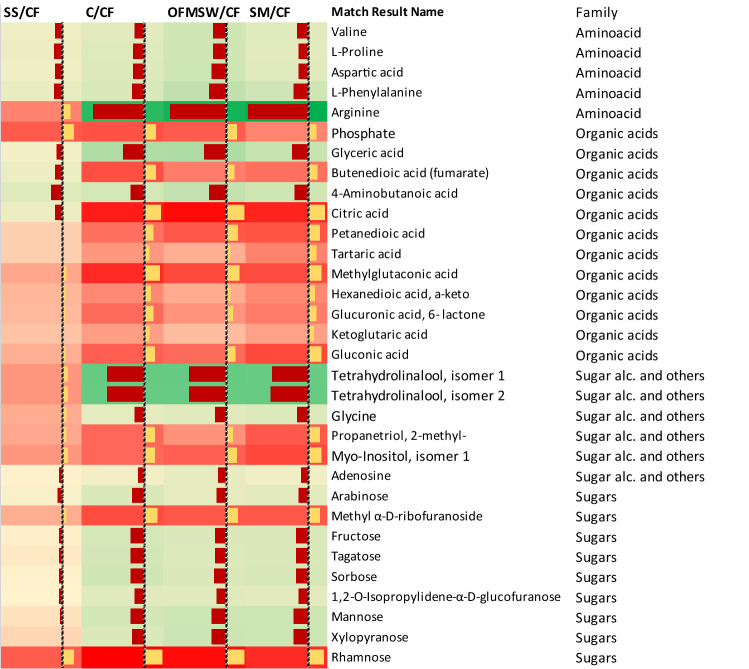
Heat map showing the log fold change ratio of the metabolites in lettuces between the different tested treatments and the chemical fertilization (CF) used as control. Only metabolites in which the comparison between treatment and control is statistically significant for at least one treatment are shown (*p *value < 0.05). Plot was created with Microsoft Excel (version16.6.6, https://www.microsoft.com/).

Figure 2 shows the heatmap for the most relevant metabolites in the different treatments. The CF was used as a reference since it is the agronomic situation in which nutrients are most easily available as well as the treatment which has the lowest occurrence of metals and OMCs, hence the abundance of each metabolite in each treatment was divided (standardized) by its abundance in the CF. This helps to show the changes in the lettuce metabolic profile due to the different fertilization products. No statistical changes were observed between the SS fertilizer and CF treatments, except for phosphate and aminobutanoic acid (GABA). In contrast, the C, OFMSW and SM treatments resulted in positive or negative statistical changes for all the shown metabolites. The heatmap clustered the samples in two groups, SS/CF (group I) and all other amendment strategies (group II). The results indicate that lettuces amended with SS and CF showed higher levels of amino acids than those amended with OFMSW or SM or not amended at all (C), especially for arginine (*p* value < 0.05). This is consistent with the fact that among the 21 proteinogenic amino acids, arginine has the highest nitrogen-to-carbon ratio, making it especially suitable as a storage form of organic nitrogen^37^. Its lower presence in these lettuces was thus consistent with the low nitrogen availability in these three treatments (Table 1), as well as the low plant growth (Fig. 1). Conversely, the relative abundance of organic acids such as fumarate and gluconic acid was greater in lettuces amended with the C, OFMSW, and SM treatments. Fumarate is directly involved in the citric acid cycle (TCA). This may indicate that under low nutrient availability, the activity of the TCA cycle was up-regulated. This is consistent with the fact that TCA is a central metabolic hub necessary for ATP production and for providing precursors used in many biosynthetic pathways^38,39^. The high abundance of gluconic acid may also be related to plant stress due to nitrogen availability. For instance, Kempa et al.^40^ observed that salinity stress in *Arabidopsis Thalina* increases the levels of gluconic acid, whereas Degenkolbe et al.^41^ observed the induction of gluconic acid in rice plants under draught stress, suggesting that deficient nitrogen availability may involve similar metabolic pathways. The reduction of the GABA levels in all treatments compared to the CF treatment seems contradictory since it has been described as a non-protein amino acid that accumulates in plant tissues in response to biotic and abiotic stress and regulates plant growth^42^. Among the “sugar alcohols and other” compound group, tetrahydrolinalool isomers showed the highest reduction in abundance in lettuces amended with the C, OFMSW, and SM treatments compared to CF, but no changes when SS fertilization was used. Linalool is an acyclic monoterpene, described as an important odorous constituent in a series of plant aromas^43^. Therefore, the results indicate that the production of this secondary metabolite only increased when plants grew without any nutrient limitations (CF and SS), allowing them to use energy for the production of secondary metabolites. On the other hand, myoinositol levels increased under low nutrient conditions (C, OFMSW, and SM). Myoinositol derivatives are involved in a large number of cellular processes, such as biogenesis of the cell wall and membrane structures, phosphate storage, cell signaling, and cell resistance to external stress factors^44^. For example, myoinositol is involved in plant tolerance to salt and cold stress^45^. Finally, the results show that the levels of certain sugars (arabinose, fructose, tagatose, sorbose, mannose, and xylopyranose) increased in lettuce plants after they were amended with the C, OFMSW, and SM treatments compared to lettuces fertilized with CF and SS. This may indicate that lettuces grown under proper fertilization are more prone to accumulate those sugars. All these carbohydrates are directly or indirectly derived from photosynthesis^46^. The only exception was rhamnose, which showed the lowest abundance in lettuces grown with the CF amendment treatment. This is consistent with the fact that rhamnose is a deoxy-sugar present in plant cell-wall pectic polysaccharides (mainly rhamnogalacturonan I and rhamnogalacturonan II), but also in diverse plant secondary metabolites^47^. Hence, its increase in abundance may be due to up-regulation in response to nutrient stress (nitrogen availability). Our results indicate that the greater concentration of metals in SS and OMCs in SM and SS did not imply changes in metabolic response, probably due to the low plant uptake.

### Pathway mapping and functional annotation

**Figure 3:**
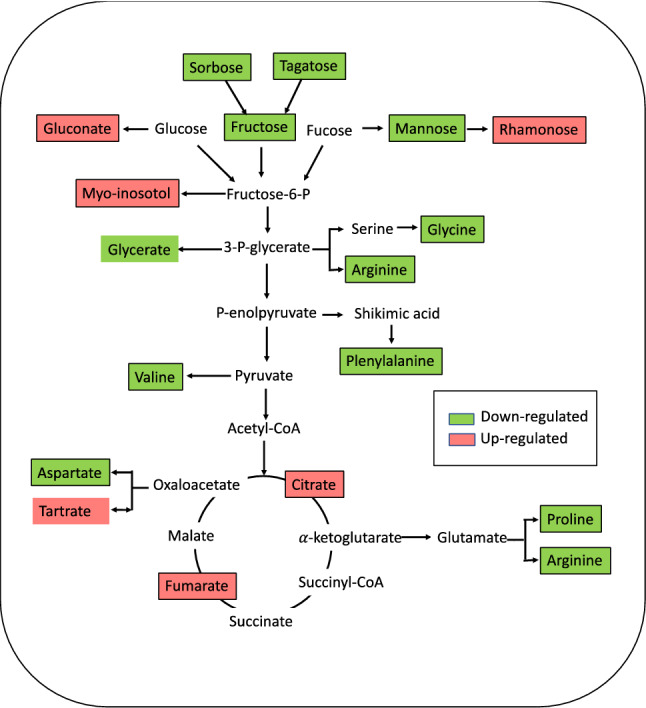
Metabolic changes involved in the lettuce crops amended OFMSW and SM fertilizers in comparison with control crops amended with CF. The significantly up- and down-regulated (*p* < 0.05) metabolites are indicated in red and green respectively. Figure was created with Microsoft PowerPoint (version16.6.6, https://www.microsoft.com/).

Primary metabolism plays an essential role in plants’ survival and development^48^. Carbon metabolism (sugar metabolism, glycolysis, TCA cycle, etc.) is essential for the production of energy and carbon skeleton compounds during growth and development^49^. The lettuces grown with the OFMSW and SM amendments or without fertilization (C) showed lower levels of different sugars (sorbose, tagatose, and mannose) and other metabolites involved in carbon metabolism (Fig. 3) compared to those grown with CF. This may indicate that the use of low-nutrient fertilizers (C, OFMSW, and SM) promotes carbon assimilation, whereas the use of high-nutrient-content fertilizers promotes carbon accumulation. This is consistent with results obtained in previous studies in which stress conditions shifted plant metabolism from carbon accumulation to carbon assimilation^14^. Conversely, other sugars (gluconate and rhamnose) showed greater concentration levels under low-nutrient conditions, probably due to their involvement in plant nutrient stress response. Nitrogen metabolism is a basic physiological mechanism for the synthesis and decomposition of nitrogenous compounds in plants^50^. From this point of view, the lower levels of amino acids in lettuce plants amended with the OFMSW and SM fertilizers compared to the CF- and SS-amended plants may be associated with the lower plant growth under these conditions. This is consistent with another study that has shown that hydroxyproline may be associated with cell elongation during plant growth, and that greater concentrations of proline and aspartic acid result in longer leaf widths and lengths^51^. The lower concentration of glycine in lettuces amended with low-nutrient-content fertilizers is associated with the lower photorespiration activity in these plants compared to plants amended with CF and SS^52^. Finally, the results show that low-nutrient-content fertilizers resulted in an increase in fumarate and citric acid, two components of the TCA. Therefore, the present results suggest that nutrient stress (available N) induced a down-regulation of amino-acid synthesis and an upregulation of the TCA cycle. These metabolic changes are completely different from those found under drought stress. For example, Zhang et al.^53^ observed that the abundance of the TCA cycle intermediates citric and fumaric acid was materially reduced by drought stress in *Caragana korshinskii*, implying that both the TCA cycle and glycolysis were inhibited, but the synthesis of almost all of the various amino-acids appeared to have been enhanced. Hence, the metabolic stress response depends on the source of stress.

**Figure 4:**
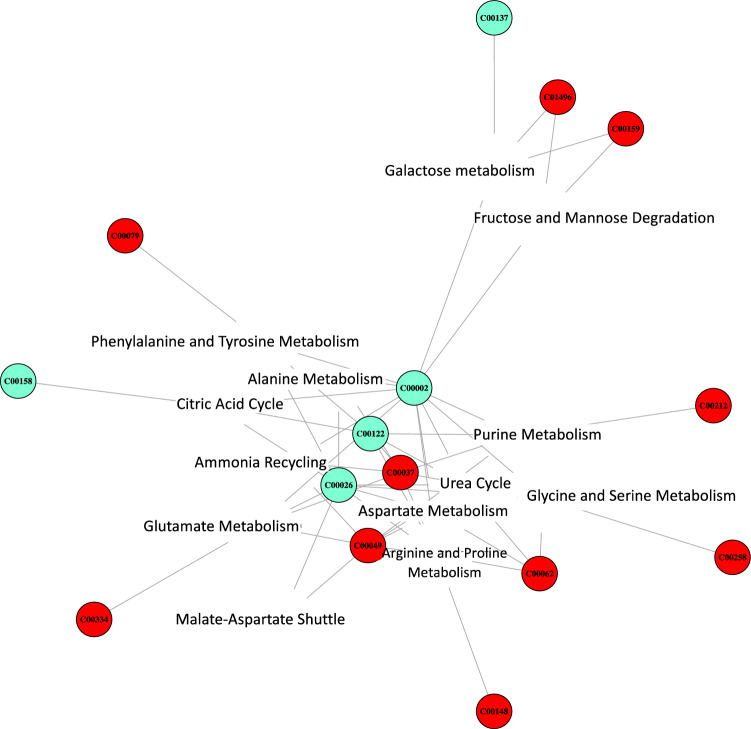
Network analysis of the metabolites and metabolic subsets identified by the MetaboAnalyst 4 software. Red and cyan circles correspond to metabolites which concentrations increase or decrease in plants grown in ammonium-rich amendments, respectively. Metabolites are identified by the corresponding KEGG code. Plot was created with MetaboAnalyst 4.0 software (https://www.metaboanalyst.ca/faces/home.xhtml).

MetaboAnalyst analysis identified 13 metabolite sets significantly enriched in metabolites differentially affected by the amendments (see Supplementary Table S3 online). Functional annotation of the enriched metabolite sets indicated that they were related to different metabolic pathways, including the urea cycle, ammonia recycling, and the metabolism of several amino acids, mainly arginine, proline, aspartate, glutamate, phenylalanine, and tyrosine, among others. In addition, two of these sets were associated with specific sugar metabolic pathways. Most of the identified sets shared a substantial fraction of detected metabolites, as revealed by the network in Fig. 4. This network shows the central position of pathways related to ammonium metabolism, including arginine metabolism, ammonium recycling, and the urea cycle, which shared metabolites with all the amino acid and central metabolism-related sets. We concluded that the corresponding metabolic changes may reflect the adjustment of the plant metabolism to high- (CF and SS) or low-ammonium growth conditions (C, OFMSW, and SM), whereas the greater concentration of metals and OMCs in SS and SM did not play an important role.

The network shown in Fig. 4 also shows that two metabolic sets related to galactose and fructose and mannose metabolism appear as an independent group, connected only to the main metabolic subset by ATP (C0002 in Supplementary Table S3 online). Fructose and mannose, together with other sugars (arabinose, sorbose, tagatose, ß-D-xylose, rhamnose), are known components of the cell wall, a specific metabolic subset not included in the MetaboAnalyst analysis since it is not specifically related to plant metabolism. The fact that the concentrations of these sugars (except rhamnose) increased in plants grown in ammonium-rich amendments may therefore be related to their faster growth rate compared to the controls. We thus concluded that most of the observed effects on metabolism concentrations related to the different amendments may be related, first, to the abundance or limitation of ammonium available to the plant and, second, to the differential growth rates of plants growing in low- or high-ammonium conditions.

In summary, the present results show that the greater concentration of heavy metals and/or OMCs in the SS and SM fertilizers did not affect lettuce phenotype and metabolism compared to the chemical fertilization, but fertilization products with low-ammonium and nitrate content fertilizers (OFMSW, SM, and no fertilization) disrupted carbohydrate and nitrogen metabolism compared to high-ammonium content fertilizers (CF and SS).

## Methods

### Experimental design

The study was conducted in a greenhouse located in the agricultural experimental station (Agròpolis) belonging to the Polytechnic University of Catalonia (UPC, Viladecans, Spain) in fall (October 3 to December 4, 2018). The average temperature inside the greenhouse was 18 °C, and the relative humidity was 56%. The experimental units consisted of 2.5 L cylindrical amber-glass pots (Ø = 15 cm, 20 cm high) filled with 2.3 kg of soil sieved to 2 mm. The soil used was collected from the agricultural field located in the nearby Agròpolis experimental station. The soil had a loam-clay texture (40% sand, 35% silt, 25% clay), a pH of 8.4, and an electrical conductivity of 0.24 dS m^−1^. The total organic carbon content was 1.27%, and the nitrogen content (Kjeldahl) was 0.09% of the soil dry weight. The Olsen phosphorous concentration was 0.033 g Kg^−1^, whereas the K^+^, Ca^2+^, Mg^2+^, and Na^+^ cations were 0.344, 7.014, 0.362, and 0.091 g Kg^−1^ of the soil dry weight, respectively.

The experimental set-up consisted of five treatments with five replicates (25 experimental units in total), SS (primary and secondary sludge with anaerobic digestion collected from a domestic WWTP), OFMSW (municipal organic food waste composted with wood residues), SM (swine manure, solid fraction), chemical fertilization (CF) as a control, and a control without fertilization (C). These two controls were set to assess the effect of nutrients on plant phenotype. Chemical fertilizer composition was as follows: ammonium nitrate (34% N), phosphate (44% P_2_O_5_) and potassium sulfate (52% K_2_SO_4_). The theoretical values of nutrients to be added (NPK) were based on previously reported studies for lettuce crops (80–100 kg of N per ha; 30–50 kg of P_2_O_5_ per ha; and 160–210 kg K_2_O per ha)^54^. Since K was already present in the soil, and the P values were greater than N for all the studied fertilizers, N was selected as the limiting nutrient (Table 1). The amount of fertilization product added per pot was calculated to ensure the same quantity of total nitrogen in all treatments (100 kg of N per ha).

4-week-old Batavia lettuce (*Lactuca sativa* L. cv. Maravilla de Verano) seedlings provided by Lladó plant nursery were planted in pots and watered with groundwater.

About 100 mL of irrigation water was applied to each experimental unit per day. The number of daily irrigations was regulated to keep available water holding capacity (the portion of the water that makes up water holding capacity and that is available to plant roots).

### Sampling strategy

Grab samples from soil and organic fertilizers were collected at the beginning of the experiment. After 60 days, leaf samples were collected in bulk from 25 experimental units on middle- and old-stage leaves using an 8-mm leaf punch disk and were immediately frozen in liquid nitrogen (10 leaf samples per pot) following a previously described sampling strategy for studying plant metabolomics^55^. Samples were stored at − 80 °C until analysis. Immediately after metabolic sampling, lettuces were harvested, weighted and measured for the leaf height and number of leaves.

### Analytical Methodologies

#### Physicochemical characterization of the soil and organic fertilizers

The chemical characterization (heavy metals and soil physicochemical characteristics) of the soil and the different fertilizer amendments was carried out at the Eurofins agro-environmental accredited laboratory (https://www.eurofins.es/).

Ultrasonic batch extraction followed by a high-performance liquid chromatography (LC)-Orbitrap mass spectrometry analysis was used for the detection of OMCs such as CECs in the organic fertilizer matrices following a previously described methodology^56^. Briefly, 500 mg of homogenous sample was extracted with 4 mL of McIlvain-EDTA buffer and 1 mL of ACN in an ultrasonic bath for 15 min. After sonication, 2 mL of lead acetate solution was added, and the sample was vigorously shaken by hand. After centrifugation, the supernatant was diluted by adding 13 ml of 0.2 McIlvain-EDTA solution to perform the SPE clean-up step. The SPE cartridges (Strata-X RP cartridge, 200 mg/6 mL) were first preconditioned with 5 mL of methanol and 5 mL of water. After sample loading, the polymeric cartridge was washed with 5 mL of water and dried under a nitrogen stream. This was followed by elution with 5 mL of methanol. The resulting eluate was evaporated to dryness and reconstituted in 200 µL of a mixture of water and ACN (97:3, v:v). The final extracts were filtered through a 0.22 µm pore nylon filter prior to injection. A Q-Exactive Orbitrap HCD (Thermo Fisher Scientific, Bremen, Germany) mass spectrometer equipped with a heated electrospray source, a Surveyor MS Plus pump, and an Accela Open Autosampler (Thermo Fisher Scientific, San Jose, California) were used for the analysis. Further information on the identification and determination of CECs by HPLC-Orbitrap can be found in the Supplementary Material section.

The description of the sample preparation steps and the UPLC-MS/MS methodologies for the determination of antibiotics in fertilization products an crops are described in Margenat et al.^23^.

#### Metabolomic analysis of lettuce leaves

The extraction procedure for the non-target analysis of metabolites in lettuce samples was adapted from a previous procedure^14^. Briefly, 10 mg of plant material was transferred to an Eppendorf tube, and 400 µL of methanol was added. Then, 30 ng of succinic acid-2,2,3,3-d_4_, -L-Serine-1-^13^C, D-Mannose-1-^13^C, D-Ribose-1-^13^C, D-Glucose-^13^C, and salicylic acid-d_6_ in methanol solution were added to the tube as internal standards to follow the extraction procedure. Samples were vortexed and sonicated in an ultrasonic bath (35 kHz) for 15 min at 37 °C. 200 µL of chloroform was then added and the samples were vortexed for 1 min and sonicated for 15 min. Next, 400 µL of water was added, and the samples were again vortexed for 1 min and sonicated for 15 min. The tubes were then centrifuged at 10,000 × g for 15 min in order to separate the aqueous and lipid phases. Finally, 700 µL of the aqueous phase was transferred to a 4 mL glass vial. The extracts were vacuum-dried with a SpeedVac (Thermo Scientific, Bremen, Germany) at 40 °C for 4 h. The samples were stored at − 80 °C until analysis for sample preservation. 80 µL of 20 mg mL^−1^ methoxyamine (MeOX) in pyridine was added to the dry residue. The mixture was vortexed for 1 min and then incubated at 30 °C for 90 min. Thereafter, 50 µL of N-methyl-N-(trimethylsilyl) trifluoroacetamide (MSTFA) with 1% trimethylchlorosilane (TMCS) was added, and the mixture was vortexed for 1 min and incubated at 37 °C for 30 min. Finally, the extracts were filtered through a 0.22 µm filter (Ultrafree-MC, Millipore) and then transferred to a chromatographic vial. Triphenylamine (TPhA) was added as an instrumental standard (25 µL), and 2µL of samples were injected into the GC-Orbitrap system (Q Exactive GC, Thermo Scientific, Bremen, Germany), which was operated in the full-scan mode and equipped with a 30 m × 0.25 mm I.D., 0.20 µm film thickness Sapiens-X5.MS coated with 5% diphenyl 95% dimethylpolysiloxane from Teknokroma (Sant Cugat del Vallès, Spain). The GC injector temperature was set at 280 °C. The oven temperature was set at 70 °C and increased to 100 °C at a rate of 7 °C/min, then to 260 °C at a rate of 5 °C/min, and finally to 300 °C at a rate of 10 °C/min. The applied electron ionization energy was 70 eV, and the transfer line and ion source were set at 280 °C and 260 °C respectively. Scanning was performed from 50 to 650 m/z at a mass resolution of 60,000 (full width at half maximum (FWHM) at m/z 200) for non-target analysis of lettuce sample extracts. Xcalibur 4.1 and TraceFinder 4.0 software (Thermo Scientific, Bremen, Germany) were used for data-processing (peak alignment and deconvolution). A signal-to-noise ratio cutoff of 200 and a mass error tolerance of 5 ppm were used to eliminate background interferences. This tool allowed peak detection with spectral deconvolution and tentative compound identification against the NIST library (mainlib and replib) for the candidate compounds. Similarity and reverse indexes (SI/RI) were used. As a general guide, a value of 900 or greater was considered a very good match; between 800 and 900, a good match; between 700 and 800, a fair match; and less than 600, a poor or very poor match (see Supplementary Table S1 online). The recoveries for the added internal standards ranged from 60 to 90%, with a repeatability (% RSD) of better than 20%.

#### Plant phenotype

The lettuce phenotype was measured at the end of the experiment for each of the studied scenarios taking into account the lettuce weight, length and number of lettuce leaves, as well as lipid and carbohydrate content^57^.

### Data analysis

Semi-quantitative non-target analysis for each metabolite was as follows: the area of the most abundant m/z was automatically integrated and normalized by the area of the m/z of the instrumental standard. This resulted in a data matrix of relative abundances for more than 300 metabolites for each of the studied fertilization conditions (SS, OFMSW, SM, CF, and C). Differences between agronomical parameters were determined by the Kruskal–Wallis test using IBM SPSS v25 software. The determination of the effect of the different cultivation treatments on the metabolite profile and morphology of the lettuce leaves was performed by multivariate statistical analysis including a partial least-squares regression analysis (PLS-RA) of the data matrix obtained from the GC-Orbitrap using Xlstat software. The effect of metabolite changes due to the different treatments was visualized using a heatmap considering only metabolites that had statistical differences between treatments (*p* value < 0.05). MetaboAnalyst 4.0 (https://www.metaboanalyst.ca) was used to elucidate key functional metabolic differences due to the application of the different organic amendment solutions. KEGG data information is included in Fig. 4 and table S-3^58–60^.

### Statements on plant material

The plant material in this manuscript complies with relevant institutional, national, and international guidelines and legislation.

## Supplementary Information

Supplementary Information.
