# Supplementary material for: Metabolomic and phenotypic implications of the application of fertilization products containing microcontaminants in lettuce (Lactuca sativa)
Source: Sci Rep. 2021 May 6;11:9701. doi: 10.1038/s41598-021-89058-x (PMC8102503; doi:10.1038/s41598-021-89058-x)
Supplement: Supplementary file 1 — Supplementary Information. [file 41598_2021_89058_MOESM1_ESM.docx]

Metabolomic and Phenotypic Implications of the Application of Fertilization Products Containing Microcontaminants in Lettuce (*Lactuca sativa*)

Víctor Matamoros^1*^, Alicia María Rendón-Mera^2^, Benjamí Piña^1^, Đorđe Tadic1, Núria Cañameras^3^, Nuria Carazo^3^, and J.M Bayona^1^

1Department of Environmental Chemistry, IDAEA-CSIC, c/Jordi Girona, 18-26, E-08034, Barcelona, Spain.

2 Universidad de Antioquia

3Department of Agri-Food Engineering and Biotechnology DEAB-UPC, Esteve Terrades 8, Building 4, Castelldefels, Spain

Number of Tables: 3

***Corresponding author**: victor.matamoros@cid.csic.es

**SM-Analytical methodology for the determination of CECs in organic fertilizers**

The chromatographic separation was performed on a Kinetex C18 column (particle size 2.6 µm, ID 2.1 mm, length 50 mm; Phenomenex, Torrance, CA, U.S.A.), including a pre-column. The flow rate was 0.25 mL/min, and the injection volume was 10 µl. A binary gradient elution program using mobile phases A (acetonitrile with 0.1% formic acid) and B (water with 0.1% formic acid) was set as follows: from 0 to 3 min, isocratic, 3% A; from 3 to 15 min, 3-20% A; from 15 to 17 min, 20-95% A; from 17 to 19 min, isocratic, 95% A; from 19 to 20 min, 95-3% A; from 20 to 30 min, isocratic, 3% A. The column oven and autosampler temperatures were set at 25ºC and 5ºC, respectively.

The analyses were carried out in positive electrospray ionization (ESI+). The parameters were: spray voltage of 3.5 kV, capillary temperature of 325 °C, heater temperature of 275 °C, sheath gas (nitrogen) flow rate of 40 psi and auxiliary gas (nitrogen) flow rate of 10 (arbitrary units). The mass range was m/z 50–1,000 in both full scan and all ion fragmentation (AIF) acquisition modes. AIF as data-independent analysis was performed using Higher-energy Collisional Dissociation (HCD) fragmentation with collision energies of 10 and 60 eV. The resolution was 50,000 at a scan rate 2 Hz, the automatic gain control was 106 and set as “balanced” with a maximum injection time of 250 ms.

Tentative identification achieved in cases which molecular ion was detected with mass accuracy below 2 ppm and at least 2 qualifier fragment ions were detected. Samples were screened for 1298 suspected emerging substances. Suspect list “NORMAN Compounds in MassBank” containing the major fragments of the selected compounds was used and can be found in website of NORMAN Suspect list exchange (NORMAN Suspect list exchange: a central website to access various lists of substances for suspect screening. https://www.norman-network.com/?q=node/236). Samples were reinjected with inclusion list to record HRMS/MS spectra of the detected compounds and verify their identity by library spectral match. The data was processed with TraceFinder 3.3 EFS software (Thermo Fisher Scientific, Bremen, Germany).

|  | **Table S1.** Retention time, m/z used for the semi-quantification, and SI/RI identification of the metabolites. | | | |
| --- | --- | --- | --- | --- |
| **Peak RT (min)** | | **m/z** | **Name** | **SI/RI** |
| 11.08 | | 144.1202 | Valine, 2TMS derivative | 749/872 |
| 12.30 | | 111.0804 | Arginine, 2TMS derivative | 528/654 |
| 12.56 | | 299.0709 | Silanol, trimethyl-, phosphate (3:1) | 852/861 |
| 13.04 | | 142.1046 | L-Proline, 2TMS derivative | 758/779 |
| 13.27 | | 183.0471 | Retinoic acid, TMS derivative | 482/585 |
| 13.32 | | 184.0398 | Glycine, 3TMS derivative | 639/785 |
| 14.01 | | 293.1351 | Glyreric acid, 3TMS derivative | 796/806 |
| 14.19 | | 245.0659 | 2-Butenedioic acid, (E)-, 2TMS derivative | 603/725 |
| 14.44 | | 127.1117 | Octanoic acid, cyclobutyl ester | 611/619 |
| 15.22 | | 201.1667 | tetrahydrolinalool, isomer 1 | 616/693 |
| 15.71 | | 145.1043 | tetrahydrolinalool, isomer 2 | 621/722 |
| 15.81 | | 239.0948 | Unknown | NA |
| 18.20 | | 143.0522 | Unknown | NA |
| 18.62 | | 232.1181 | Aspartic acid, N-trimethylsilyl-, bis(trimethylsilyl) ester | 823/851 |
| 18.74 | | 174.1126 | 4-Aminobutanoic acid, 3TMS derivative | 842/845 |
| 18.86 | | 157.0678 | Unknown | NA |
| 19.24 | | 198.0579 | Pentanedioic acid, 2(methoxamyno)-TMS | 619/681 |
| 19.49 | | 169.0678 | Uridine, 3TMS derivative | 611/627 |
| 19.54 | | 95.0855 | Unknown | NA |
| 19.60 | | 219.1230 | Propanetriol, 2-methyl-, tris-O-(trimethylsilyl)- | 637/748 |
| 20.95 | | 218.1026 | L-Phenylalanine, 2TMS derivative | 662/750 |
| 21.58 | | 150.0445 | Tartaric acid, 4TMS derivative | 748/776 |
| 21.63 | | 239.0948 | Unknown | NA |
| 22.07 | | 103.0574 | Arabinose, tetrakis (trimethylsilyl)ester, methyloxime | 772/777 |
| 22.65 | | 129.0366 | Succinic acid, ethyl 4-methylhept-3-yl ester | NA |
| 22.72 | | 255.0866 | Quinnic acid, 5TMS derivative | 507/587 |
| 23.25 | | 244.1309 | Methylglutaconic acid, 2TMS | 545/603 |
| 23.30 | | 239.0948 | Dehydroabietic acid, TMS derivative | 500/548 |
| 23.39 | | 133.0680 | Inose, 2-desoxy-, O-methyloxime, tetrakis-O-(trimethylsilyl) | 653/677 |
| 23.79 | | 217.1073 | Methyl α-D-ribofuranoside, 3TMS derivative | 624/674 |
| 24.22 | | 217.0711 | Tagatofuranose, pentakis (trimethylsilyl)ether | 667/699 |
| 24.27 | | 302.1237 | Hexanedioic acid, a-keto oxime, tris-(trimethylsilyl)- | 565/586 |
| 24.43 | | 292.1340 | Ribonic acid-5TMS derivative | 775/807 |
| 24.64 | | 319.1264 | Galactose, pentakis(trimethylsilyl)ether, ethoxyme | 652/660 |
| 24.86 | | 89.0417 | Glucuronic acid, 6- lactone, 3TMS | 592/633 |
| 25.27 | | 217.1072 | Galactaric acid, 6 TMS derivative | 541/642 |
| 25.40 | | 273.0971 | Citric acid, 4TMS derivative | 780/792 |
| 25.84 | | 157.0679 | Unknown | NA |
| 25.86 | | 131.0523 | Unknown | NA |
| 25.92 | | 157.0315 | Ketoglutaric acid, 2 TMS derivative | 599/709 |
| 26.24 | | 159.0835 | Galactofuranose, 2,6-di-O-methyl-3TMS derivative | 555/631 |
| 26.72 | | 117.0365 | Fructose, TMS derivative | 773/775 |
| 26.88 | | 117.0365 | Tagatose,TMS derivative | 755/759 |
| 26.93 | | 219.1044 | Sorbose, TMS derivative | 730/745 |
| 27.25 | | 319.1573 | Galactose oxime, 6TMS derivative | 785/788 |
| 27.27 | | 217.1072 | 1,2-O-Isopropylidene-α-D-glucofuranose, 3TMS derivative | NA |
| 27.51 | | 147.0655 | Mannose, TMS derivative | 803/806 |
| 28.33 | | 318.1494 | Myo-Inositol, 6TMS derivative | 839/842 |
| 28.82 | | 117.0365 | Unknown | NA |
| 29.49 | | 292.1340 | Gluconic acid, 6TMS derivative | 624/701 |
| 29.74 | | 204.099 | Xylopyranose, 4TMS derivative | 753/791 |
| 30.81 | | 217.1072 | Myo-Inositol, 6TMS derivative | 828/830 |
| 31.60 | | 139.1118 | Unknown | NA |
| 31.86 | | 139.1118 | Unknown | NA |
| 32.01 | | 157.1222 | Allose, TMS | 731/738 |
| 32.28 | | 157.1222 | Unknown | NA |
| 32.96 | | 105.0369 | Unknown | NA |
| 34.81 | | 204.0995 | Glucopyranose, 5TMS derivative | 812/841 |
| 35.09 | | 204.0351 | Unknown | NA |
| 35.16 | | 204.0351 | Unknown | NA |
| 35.34 | | 111.0441 | Unknown | NA |
| 35.88 | | 204.0995 | Rhamonose, 4TMS derivative | 651/766 |
| 39.38 | | 236.0961 | Adenosine, 4TMS derivative | 781/799 |
| 40.50 | | 217.1072 | Sorbofuranose, 5TMS derivative | 778/805 |
| 40.68 | | 217.1072 | Psicofuranose, 5TMS derivative | 756/789 |
| 43.35 | | 204.0995 | L-Rhamnose, 4TMS derivative | 651/766 |
| 44.48 | | 204.0996 | Maltose, 8TMS derivative, isomer 1 | 769/789 |
| 46.50 | | 169.0678 | Turanose, 8TMS derivative | 752/755 |
| 47.45 | | 361.1678 | Sucrose, 8TMS derivative | 698/720 |
| 47.46 | | 204.0995 | Manobiose, 8TMS | 660/687 |
| 47.87 | | 217.1072 | Ribofuranose, 3TMS derivative | 733/796 |

Table S2. Compounds tentatively identified in organic fertilizers and their use.

| **SS** |  | **SM** |  | **OFMSW** |  |
| --- | --- | --- | --- | --- | --- |
| **Compound Name** | **Use** | **Compound Name** | **Use** | **Compound Name** | **Use** |
| Dehydroepiandrosterone | endogenous steroid hormone | 2-Naphthoxyacetic acid | Plant growth regulator | Diethyl phthalate | pharmaceutic aid (plasticizer) |
| Levofloxacin | antiobiotic | Doxycycline | antibiotic | Propiconazole | antifungal |
| N-Desmethyltramadol |  | Ibuprofen | NSAID | N,N-Bis(2-hydroxyethyl)dodecanamide | used in: washing and cleaning products, adhesives and sealants |
| Ofloxacin | antiobiotic | Flubendazole | anthelmintic |  |  |
| Citalopram | antidepressant | 2-Naphthalenecarboxylic acid |  |  |  |
| DEET | repellent | Phenylephrine | vasoconstrictor |  |  |
| Verapamil metabolite D617 |  | Imiprothrin | insecticide |  |  |
| N,N-Didesvenlafaxine |  | 5,5-Diphenylhydantoin | antiepileptic |  |  |
| MDMA | stimulant and hallucinogen | 2,4-Dihydroxybenzophenone | constituent of synthetic perfumes |  |  |
| Amisulpride | antipsychotic | Indoline |  |  |  |
| Ciprofloxacin | antiobiotic | Lignocaine N-oxide |  |  |  |
| Flecainide | antiarrhythmic | Lincomycin | antibiotic |  |  |
| Norfloxacin | antiobiotic | Tetracycline | antibiotic |  |  |
| Naptalam | herbicide | 4-Androstene-3,17-dione | steroid hormone |  |  |
| Phenylephrine | vasoconstrictor | Testosterone | replenisher (androgen) and androgen receptor agonist |  |  |
| Azithromycin | antiobiotic | Medroxyprogesterone | female hormon |  |  |
| Pioglitazone | antiemetic and prokinetic |  |  |  |  |
| Domperidone | antiemetic, gastroprokinetic agent |  |  |  |  |
| Mexiletine | antiarrhythmic |  |  |  |  |
| Paroxetine | antidepressant |  |  |  |  |
| Clomipramine | antidepressant |  |  |  |  |
| Rimantadine | antiviral |  |  |  |  |
| 8-Hydroxyquinoline | disinfectant |  |  |  |  |
| Thiabendazole | anthelmintic and antifungal |  |  |  |  |
| Clozapine | antipsychotic |  |  |  |  |
| Imazalil | antifungal |  |  |  |  |
| Miconazole | antifungal |  |  |  |  |
| Norgestrel | contraceptive and progesterone receptor agonist |  |  |  |  |
